# Effects of Combined Exercise and Low Carbohydrate Ketogenic Diet Interventions on Waist Circumference and Triglycerides in Overweight and Obese Individuals: A Systematic Review and Meta-Analysis

**DOI:** 10.3390/ijerph18020828

**Published:** 2021-01-19

**Authors:** Hyun Suk Lee, Junga Lee

**Affiliations:** 1 Graduate School of Education, Chung-Ang University, Seoul 06974, Korea; hslee@cau.ac.kr; 2 Sports Medicine and Science, Global Campus, Kyung Hee University, Seoul 17104, Korea

**Keywords:** exercise, ketogenic diet, obesity, meta-analysis

## Abstract

(1) *Background*: The purpose of this meta-analysis was to investigate the effects of combined exercise and low carbohydrate ketogenic diet interventions (CELCKD) for overweight and obese individuals. (2) *Methods*: Relevant studies were searched by using the MEDLINE and EMBASE databases up to October 2020. Study Inclusion and Exclusion Criteria: Inclusion criteria were reporting effects of the CELCKD for overweight and obese individuals from randomized controlled trials. Studies that did not match the inclusion criteria were excluded. The methods for CELCKD and outcomes of selected studies were extracted. The effect sizes for interventions that included cardiorespiratory fitness, body composition, fasting glucose, and lipid profiles were calculated by using the standardized mean difference statistic. (3) *Results*: A total of seven studies and 278 overweight and obese individuals were included. The average intervention of selected studies consisted of moderate to vigorous intensity, 4 times per week for 9.2 weeks. Participating in CELCKD interventions was decreased triglycerides (*d* = −0.34, CI; −0.68–−0.01, *p* = 0.04) and waist circumference (*d* = −0.74, 95% confidence interval [CI]; −1.28–−1.20, *p* = 0.01), while cardiovascular fitness, body composition, fasting glucose, total cholesterol, high density lipoprotein (HDL) cholesterol, and low density lipoprotein (LDL) cholesterol were not statistically different after the interventions. No adverse side effects were reported. (4) *Conclusions*: Participation in interventions by overweight and obese individuals had beneficial effects including decreased waist circumference and triglycerides. Longer term intervention studies with homogenous control groups may be needed.

## 1. Introduction

More than 1.9 billion adults and elders (about 52% of adults and elders) were overweight and obese in 2016 [1]. Obesity increased threefold from 2016 to 1975. America had an obesity prevalence of 42.4% in 2017~2018, which was an increase of 35.0% in 1999~2000 [2]. Increased populations of overweight and obese individuals were associated with increased chronic diseases including diabetics, metabolic syndrome, cardiovascular diseases, and cancer [3]. Maintaining normal weight has been recommended to help individuals live longer and healthier lives [4].

There were several guidelines that included exercise and a nutritional diet for maintaining normal weight [5]. Recommendations for exercise have included participating in regular moderate intensity aerobic exercise for at least 150 min per week or more than 300 min per week to achieve long-term weight control; weight lifting at moderate intensity two times per week with 10 to 15 resistance exercise repetitions [6]. Nutritional recommendations have included a 60% carbohydrate intake, 25% protein intake, and 15% fat intake for a balanced nutritional diet and restrictions on calorie intake [7]. Since the last couple of decades, previous studies have suggested different diet patterns including low carbohydrate ketogenic diets that limit individuals to less than 130 mg of carbohydrates per day consisting of about 60% fat intake, 25% protein intake, and 15% carbohydrate intake without caloric restrictions [8]. Limiting caloric intake during obesity was the main barrier to participation in weight loss programs. The low carbohydrate ketogenic diet did not have any caloric restrictions, which is attractive to individuals who worry about caloric intake. Additionally, restricted carbohydrate intake triggered insulin release from pancreatic β cells during limited stimulations with insulin. The low carbohydrate ketogenic diet increased insulin sensitivity and leptin sensitivity, which reduced feelings of hunger and further helped manage insulin levels. Obese individuals who experience the “yo-yo” effect, a cycle of weight loss and weight regain common in obesity, gain more weight after completing calorically restricted diets [9]. They were looking for easy and effective diets that did not limit caloric intake and improved insulin sensitivity and leptin sensitivity in individuals with metabolic disorders including diabetes and metabolic syndrome. Previous studies of exercise or low carbohydrate ketogenic diets reported reduced weight, improved body composition, and physical outcomes, respectively [10,11]. However, several previous studies reported controversial findings and there were several debates about high fat intake [12,13]. Additionally, studies of exercise or diverse diet restrictions showed limited weight loss effects for combined exercise and low carbohydrate ketogenic diet interventions and reported conflicting findings regarding weight loss, body composition, and other physical and psychological factors. While there is no meta-analysis study that included examining the effectiveness of combined exercise and low carbohydrate ketogenic diets for overweight and obese individuals, several review studies reported the effects of those combined interventions. Therefore, the purpose of this meta-analysis was to investigate the effectiveness of combined exercise and a low carbohydrate ketogenic diet for overweight and obese individuals and to provide guidelines for exercise and diet interventions for these individuals.

## 2. Materials and Methods 

### 2.1. Searching Processes to Identify Eligible Studies

The guideline for meta-analysis used was the Preferred Reporting Items for Systematic Reviews and Meta-Analyses [14]. Eligible studies were identified by searching the MEDLINE and EMBASE databases for papers published in English up to September 2020. Key words used in this search were “overweight,” “obesity,” “exercise,” “low carbohydrate,” “ketogenic diet,” “low carbohydrate,” AND “high fat,” and all possible combinations of those key words. Additionally, lists of citations from review studies were reviewed to identify any missed studies. Two researchers (J. L. and H. L) independently searched for relevant studies. We had further discussions about any disagreements during the selection process. Inclusion criteria for this meta-analysis were reported outcomes of interventions that combined exercise and low carbohydrate ketogenic diets for overweight and obese individuals, studies that investigated a combined exercise intervention and low carbohydrate ketogenic diet, reported participant body mass index (BMI), was a randomized controlled trial, and included overweight and obese individuals (BMI, >25 kg/m^2^). Exclusion criteria were review studies, pilot studies, protocol studies, and no report of outcomes due to a combined exercise and low carbohydrate ketogenic diet intervention, no participant BMI, and a protocol that did not include exercise and diet interventions. 

### 2.2. Statistical Analysis Data Extraction

Characteristics of the selected studies that included the first author’s name, year of publication, country of study, study design, sample size, exercise protocols, low carbohydrate ketogenic protocols including ketone assessments, ketone bodies values to control for diet adherence, and main outcomes, are presented in Table 1. Assessments of quality and risk of bias were rated and are presented in Supplementary Table S1 based on the Cochrane Collaboration’s Risk of Bias Tool [15]. The quality and bias risks assessment included seven domains: random sequence generation, allocation, concealment, blinding of participants and personnel, blinding of outcomes assessments, complete outcome data, selective reporting, and other biases, and each domain was assessing as low, moderate, or high risk of bias, not as a range of scores. 

### 2.3. Statistical Analysis 

The standardized mean difference statistic after completing exercise interventions is the difference between treatment and control groups means divided by the pooled standard deviation used to calculate the effect size for each outcome variable. The treatment groups consisted of exercise plus low carbohydrate ketogenic diet and the control groups of exercise plus usual diet. The effect size in this meta-analysis was calculated, when at least two studies reported the same outcomes. Effect size magnitude was interpreted as follows: small effect size was 0.2–0.5, medium effect size was 0.5–0.8, and a large effect size was >0.8. Heterogeneity among the effect sizes was determined by the Q statistic. When I^2^ was ≤50%, heterogeneity was absent using a fixed-effects model. When I^2^ was >50%, heterogeneity did exist using used a random effects model. Comprehensive Meta-Analysis Version 1.25 software (Biostatic Inc, Englewood, NJ, USA) was used to complete all analyses.

## 3. Results

**Figure 1:**
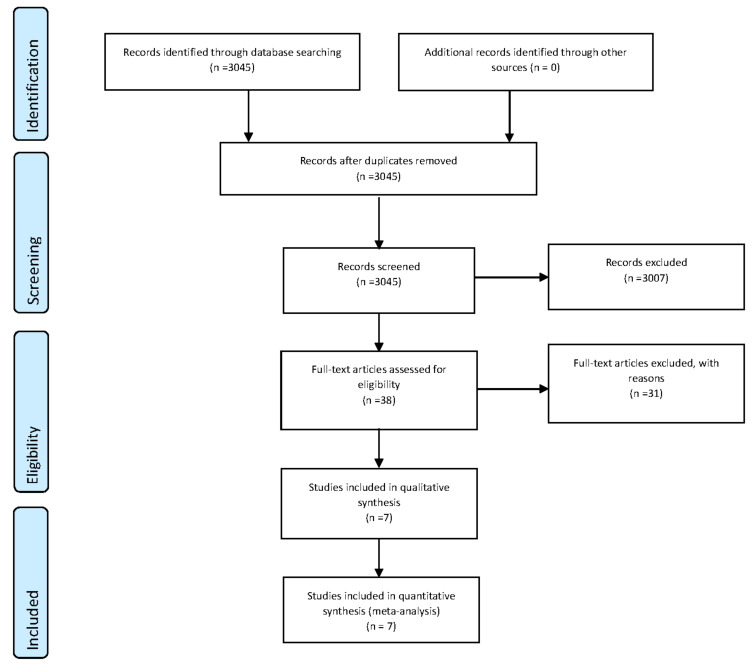
Selection process for the systematic review and meta-analysis.

The selection process for this meta-analysis is presented in Figure 1. A total of 3045 studies from the initial screening were found. The titles and abstracts from the initial screening were reviewed and 3007 studies, which were review studies, pilot studies, or not related to this meta-analysis, were excluded. Full texts of the remaining 38 studies were reviewed to determine eligibility, and finally, seven studies were selected [16,17,18,19,20,21]. A total of 255 overweight and obese individuals were included in this meta-analysis. Four studies were conducted in America, two studies in Europe, and one study in Asia. A control group of exercise and usual dietary intake was included. Quality and risk of bias assessments for selected studies were conducted using the Cochrane Collaboration’s Risk of Bias Tool, without the total scores or cutoff scores for quality and risk of bias, which maybe have produced unclear results with respect to establishing the quality and risk of bias (Supplementary Table S1). We did not find a high risk of bias when assessing the quality of risk bias. Adverse side effects for the combined exercise and low carbohydrate ketogenic diet interventions were not reported. 

### 3.1. Exercise and Ketogenic Diet Interventions

Average periods of exercise and ketogenic diet intervention ranged from 4 to 24 weeks (9.2 weeks). Three studies included high intensity interval training, two studies used moderate intensity aerobic exercise, three studies used resistance exercise, and one study used CrossFit exercise. Average frequency and duration of exercise was 4 times per week for one hour. Ketogenic diet components averaged 65% fat, 25% protein, and 10% carbohydrates and did not restrict calorie intake. The ketone levels of participants or their daily food diaries were recorded and used to assess the status of the ketogenic diet.

### 3.2. Body Compositions 

Combined exercise and ketogenic diet groups were included in 6 trials, body mass index (BMI) was included in 5 trials, % body fat mass in 3 trials, lean body mass in 2 trials, waist circumference in 2 trials. There was a statistically significant decrease in waist circumference compared to the control group (*d* = −0.74, 95% confidence interval [CI]; −1.24–−0.20, *p* = 0.01]). Body mass index (BMI) (*d* = −0.33, 95% confidence interval [CI]; −0.68–0.02, *p* = 0.67]), % body fat mass (*d* = −0.06, 95% confidence interval [CI]; −0.44–0.32, *p* = 0.78]), and lean body mass (*d* = −0.77, 95% confidence interval [CI]; −2.45–0.90, *p* = 0.37]) showed not statistically significant effect sizes post-intervention compared to control groups.

### 3.3. Cardiorespiratory Fitness (VO_2peak_)

**Figure 2:**
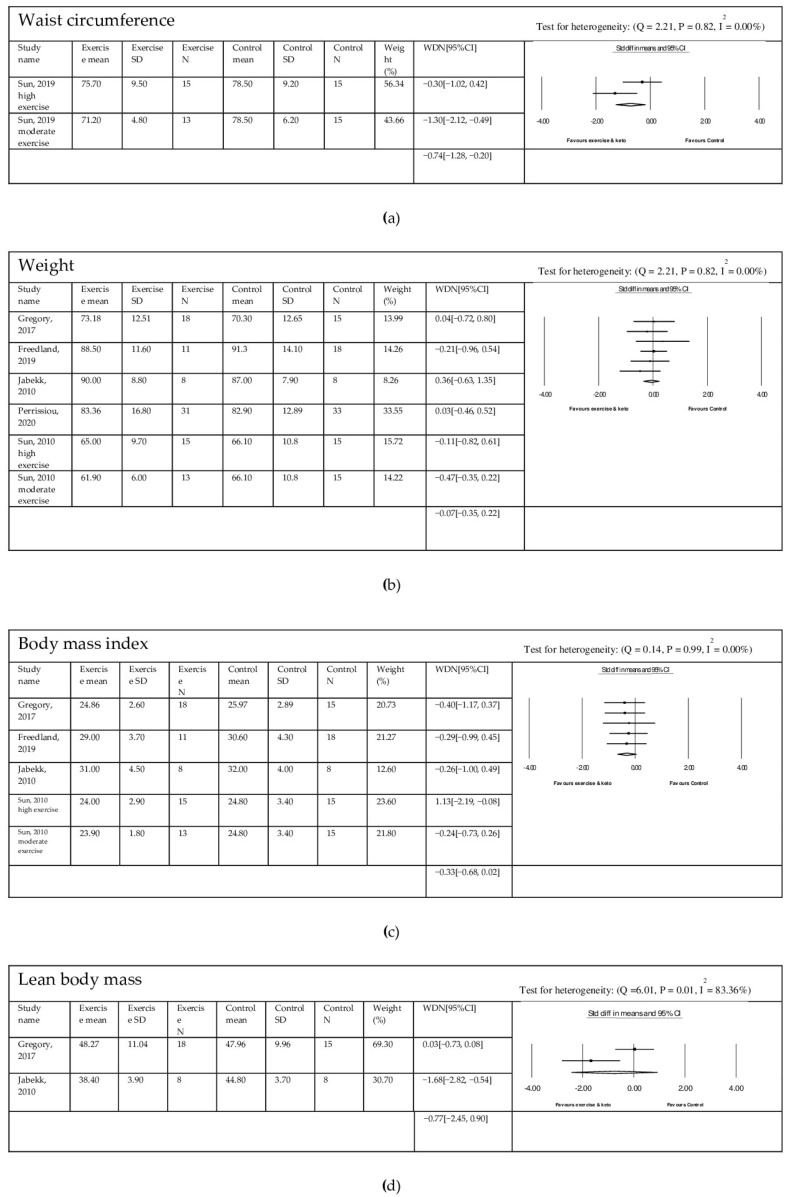

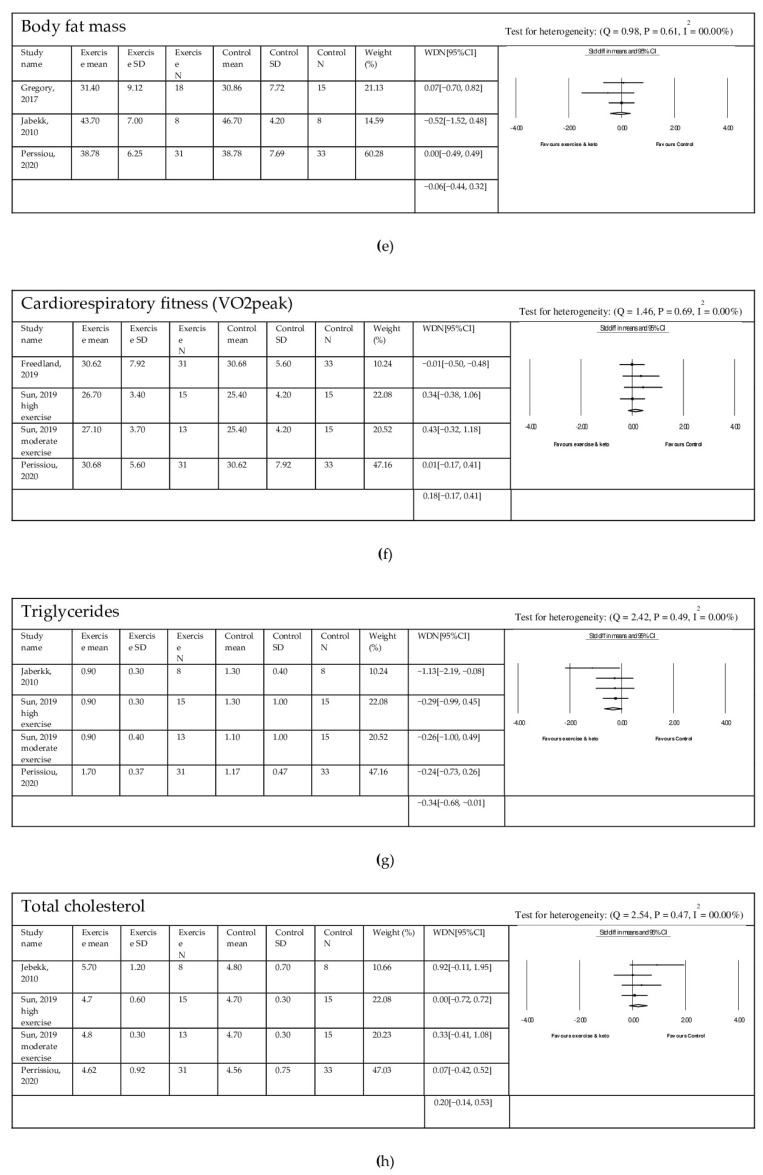

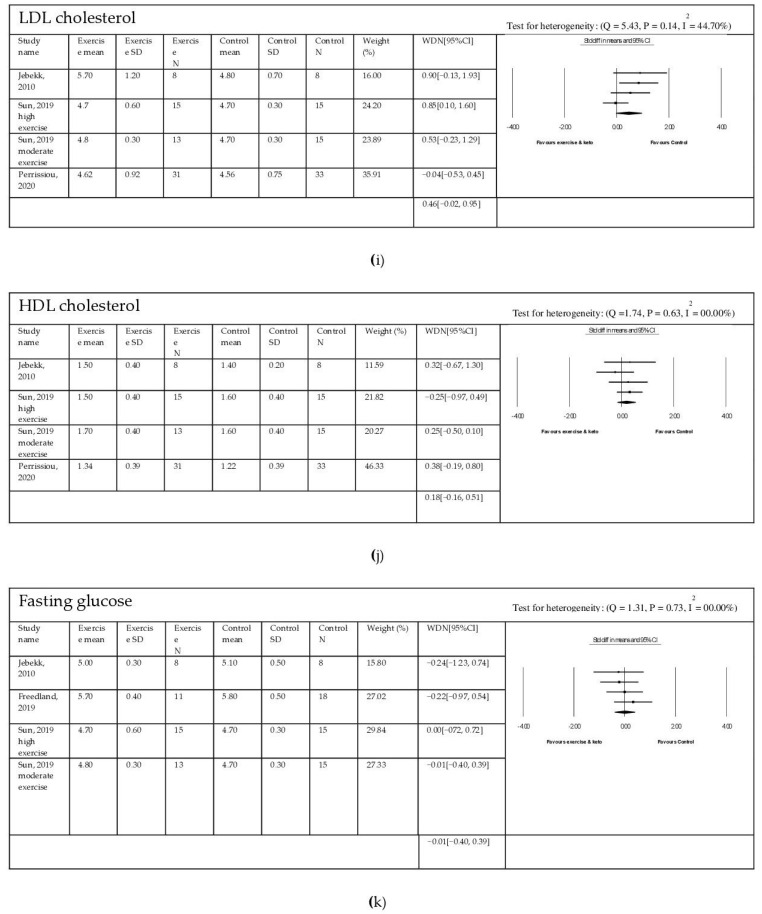
Effect sizes of exercise and ketogenic diet intervention for overweight and obese individual: (**a**) Effects of combined exercise and low carbohydrate ketogenic diet interventions on waist circumference, (**b**) Effects of combined exercise and low carbohydrate ketogenic diet interventions on weight, (**c**) Effects of combined exercise and low carbohydrate ketogenic diet interventions on body mass index, (**d**) Effects of combined exercise and low carbohydrate ketogenic diet interventions on lean body mass, (**e**) Effects of combined exercise and low carbohydrate ketogenic diet interventions on body fat mass, (**f**) Effects of combined exercise and low carbohydrate ketogenic diet interventions on Cardiorespiratory fitness (VO_2peak_), (**g**) Effects of combined exercise and low carbohydrate ketogenic diet interventions on Triglycerides, (**h**) Effects of combined exercise and low carbohydrate ketogenic diet interventions on total cholesterol, (**i**) Effects of combined exercise and low carbohydrate ketogenic diet interventions on LDL cholesterol, (**j**) Effects of combined exercise and low carbohydrate ketogenic diet interventions on HDL cholesterol, (**k**) Effects of combined exercise and low carbohydrate ketogenic diet interventions on fasting glucose.

Subjects who participated in 4 trials of combined exercise and ketogenic diet did not have a statistically significant increase in cardiorespiratory fitness (*d* = 0.18, 95% confidence interval [CI]; −0.17–0.41, *p* = 0.43]) compared to the control groups (Figure 2).

### 3.4. Fasting Glucose and Lipid Profiles

Combined exercise and ketogenic diet groups in 4 trials had statistically significantly decreased triglycerides (*d* = −0.34, 95% CI; −0.68–−0.01, *p* = 0.04) compared to the control groups. Fasting glucose (*d* = −0.01, 95% confidence interval [CI]; −0.40–0.39, *p* = 0.98]), total cholesterol (*d* = 0.20, 95% confidence interval [CI]; −0.14–0.53, *p* = 0.25]), low-density lipoprotein (LDL) (*d* = 0.46, 95% confidence interval [CI]; −0.02–0.95, *p* = 0.65]), and high-density lipoprotein (HDL) (*d* = 0.18, 95% confidence interval [CI]; −0.16–0.51, *p* = 0.31]) were not statistically significant post-intervention compared to control groups.

## 4. Discussion

Overweight and obese individuals in combined exercise and low carbohydrate ketogenic diet interventions had beneficial effects post-intervention that included decreased waist circumference and triglycerides compared to control groups. Body composition metrics, fasting glucose, and lipid profiles were not statistically different post-intervention compared to control groups. While more studies may be needed, the effects of combined exercise and ketogenic diet interventions can help to improve body composition and triglycerides. The reduction in waist circumference and triglycerides without body mass and body composition changes was clinically meaningful because waist circumference and triglycerides are inversely associated with risks of chronic disease [22,23]. 

Participating in combined exercise and low carbohydrate ketogenic diet interventions helped reduce waist circumference in overweight and obese individuals. No meta-analysis of these effects has been reported previously, and several individual previous studies were conflicting [17,18]. The current meta-analysis found decreased waist circumference in the combined exercise and low carbohydrate ketogenic diet intervention groups compared to control groups. Nevertheless, more studies are needed to confirm these results due to the limited sample size in this meta-analysis study. Decreased waist circumference was meaningful because it has an inverse association with chronic diseases [22,24]. Compared to control groups in the current meta-analysis, body composition metrics including weight, BMI, % body fat, and lean body mass were not statistically different post-intervention. These results may be clearer when more studies are conducted and homogeneous control groups are included, no treatments included exercise interventions with standard diet as control groups. Additionally, other measurements of body composition including visceral fat and other ectopic fats using computed tomography (CT), magnetic resonance imaging (MRI), volume-localized 1H-magnetic resonance spectroscopy (MRS), and dual-energy X-ray absorptiometry (DAX) are needed to assess body composition metrics that provide more specific results. 

Cardiorespiratory fitness was increased after combined exercise and low carbohydrate ketogenic diet interventions when compared to control groups, but this effect was not statistically significant. One reason for this lack of statistically significant effect is because participants in both the intervention groups and control groups included exercise and the only difference between the groups was whether the diet was a low carbohydrate ketogenic diet or a usual diet. Additional studies are needed to make proper conclusions about the intervention effects of exercise plus a low carbohydrate ketogenic diet as the experimental group and low carbohydrate ketogenic diet only as the control group. A recent meta-analysis reported that higher cardiorespiratory fitness among overweight and obese individuals was related to a lower incidence of metabolic syndrome compared to individuals with lower cardiorespiratory fitness [25]. Improving cardiorespiratory fitness is important for overweight and obese individuals to reduce the incidence of chronic diseases which may be affected by exercise rather than diet.

Overweight and obese individuals in the combined exercise and low carbohydrate ketogenic diet intervention groups showed the favorable effect of reduced triglycerides. Current findings for decreased triglycerides are supported by several previous studies [20,26]. An aerobic exercise training intervention in obese participants showed decreased hepatic and visceral lipids including triglycerides without weight loss [27]. Additionally, obese individuals who had a ketogenic diet showed decreased circulating triglycerides independent of significant weight loss [28]. The combined effects of exercise and ketogenic diet showed reduced triglycerides in overweight and obese individuals. Current findings did not show statistically significant changes in other lipid profiles, including total cholesterol, LDL, and HDL. More studies are needed since other findings from previous studies were controversial [17,19,20]. While our findings regarding lipid profiles did not show statistically significant changes, a previous study reported a significantly decreased lipid profile. The findings of other previous studies coincided with our findings. While there are several debates regarding high fat intake that may be associated with increased risks of cardiovascular diseases [29], the selected studies in the current meta-analysis did not report any adverse side effects. The average length of the combined intervention with exercise and low carbohydrate ketogenic diet in our meta-analysis was 9.2 weeks. Longer-term studies of the interventions including exercise and high fat diets need to occur to confirm side effects. 

The exercise interventions in our meta-analysis were moderate to vigorous aerobic and resistance exercise, at an average of 4 times per week, and more than 60 min per day for 9.2 weeks. The average length of the exercise interventions agreed with the recommended guidelines of the American College of Sports Medicine for obesity. These guidelines recommend moderate to vigorous aerobic activity starting with a minimum of 30 min per day, increasing to 60 min per day, and including resistance exercise, flexibility exercises, and activities of daily living. In addition, the low carbohydrate ketogenic diet consisted of low carbohydrate intake (<50 g/day, <10% of energy), high fat intake (>65% of energy), and protein intake (>25% of energy). The length of the low carbohydrate ketogenic diet interventions in the selected studies ranged from 4 to 24 weeks. An initial period of 2 weeks in the low carbohydrate ketogenic diet allowed the subjects to adapt to the new diet and reveal several side effects such as dizziness, fatigue, and stomach pain. The severity of these side effects was low enough to not warrant extensive study in future research.

There were several strengths and limitations to this current meta-analysis. This meta-analysis is the first meta-analysis to report the effects of combining exercise and low carbohydrate ketogenic diet in overweight and obese individuals from randomized controlled trials by analyzing effect sizes of the outcomes of the interventions. In addition, we are presenting in detail effective exercise programs from the selected studies as exercise guides for overweight and obese individuals. There were several limitations in this meta-analysis. First, the number of selected studies was too small to clarify current findings, even though all relevant studies were searched. Second, participants in the control groups in the selected studies underwent exercise interventions with usual diet. The exercise interventions in the control groups were not systematically controlled or designed in a way that let the subjects participate in their usual physical activity and exercise or the same exercise interventions as the experimental groups. This participant sample was small such that our results may have underestimated the effects of the combined interventions. Third, a total of 278 participants were included in this meta-analysis, and many more participants are needed to allow for a wide generalization of our findings. Only one study was conducted in Asia, which shows the need for more diverse cohorts. Lastly, the length of the exercise and low carbohydrate ketogenic interventions was an average of 9.2 weeks except for one study, which lasted 6 months. Longer-term studies of these combined interventions may be needed to estimate long-term effects for overweight and obese individuals. 

The beneficial effects of combined exercise and low carbohydrate ketogenic diet may be associated with several possible mechanisms. Exercise training that includes aerobic and resistance training for overweight and obese individuals may enhance mitochondrial function including increased mitochondrial volume and protein turnover due to damaged protein degradation and new functional protein synthesis, skeletal muscle changes that include increased metabolic enzymes, ratio of capillaries to muscle fibers, insulin sensitivity, decreased catabolic mRNA expression, cardiac muscle changes including increased contractility and relaxation and decreased left ventricular stiffness, and conduit artery changes that include increased antioxidant expression, manganese, and decreased prooxidant expression that result in increased cardiovascular fitness [30]. Low carbohydrate ketogenic diets increase fat utilization due to enhanced metabolism due to carbohydrate to fat oxidation [31,32], higher HDL, and decreased triglycerides [33,34,35]. In addition, increased exercise intensity increases stimulation of muscle hormone sensitive lipase that leads to increased intra-muscular triglyceride hydrolysis [36,37]. 

A recommendation for a proper design regarding exercise and ketogenic diet is a randomized controlled trial (RCT) because this design helps reduce bias in the treatment effects by randomly allocating subjects to more than two groups including control and experimental groups. Additionally, the control groups need to use interventions of usual diet plus exercise and usual diet only without exercise. Seven of the selected studies, found based on the inclusion and exclusion criteria, were randomized controlled trials that used combined exercise and low carbohydrate ketogenic diet as experimental groups and usual diet plus exercise as the control groups. A previous study investigated the effects of a low carbohydrate ketogenic diet versus a control with a low fat diet to reduce fatness and hyperlipidemia, while the previous study did not include exercise interventions [12]. More high quality randomized controlled studies may be needed to produce stronger evidence of the combined effects of exercise and ketogenic diets.

## 5. Conclusions

Overweight and obese individuals who regularly engage in “yo-yo” dieting regimens and suffer from metabolic disorders need effective weight loss programs other than and continuously counting their caloric intake in every meal. This meta-analysis found effective interventions for these individuals. Overweight and obese individuals who participated in a combined exercise and low carbohydrate ketogenic diet intervention, had favorable effects of waist circumference and triglycerides post-intervention, but the body composition metrics, fasting glucose, and other lipid profiles were not statistically different post-intervention in the current meta-analysis. Additional studies are needed to confirm these findings due to the limited number of randomized controlled trials included, inconsistent control groups, and restricted measurements for each component including body composition metrics. Average exercise interventions in current meta-analysis were 9.2 weeks, moderate to vigorous intensity exercise, at 4 times per week, and 60 min per day of aerobic and resistance exercise with low carbohydrate ketogenic diet interventions. While more studies regarding longer term training in the combined exercise and low carbohydrate ketogenic diet interventions may be needed, the interventions for overweight and obese individuals in the current meta-analysis did not report any adverse side effects. 

## Figures and Tables

**Table 1:** Basic characteristics of selected studies.

First Author (Year), Country	Design, Number of Participants Per Group, Age	Exercise Intervention	Ketogenic Diet	Outcomes
Gregory (2017), USA	RCT: exercise + ketogenic diet (*n* = 12) vs. control (*n* = 15), 34.58 ± 9.26 years old	6 weeks, 4 CrossFit workouts/week	6 weeks, restricting carbohydrate intake to no more than 50 g/week (<10% of energy), given a detailed guide on acceptable low-carbohydrate, fat, protein rich foods, keeping dietary food intake records, urinary ketone: ketosis > 15 mg/dl	Body composition (weight, body mass index (BMI), body fat, fat mass, lean body mass), performance (vertical jump, standing long jump, total performance time)
Gyorkos (2019), USA	RCT: exercise + carbohydrate-restricted Paleolithic-base diet (*n* = 12), vs. control (*n* = 12), 40.9 ± 20.2 years old)	4 weeks, high intensity interval training 10 × 60 s cycling intervals and 60 s of recovery, 3 days/week	4 weeks, carbohydrate-restricted Paleolithic based diet, protein (25%), fat (60%), carbohydrate (15%, 50 g/d), no restricted calories, blood ketone ß-hydroxybutyrate ketone level: 0.53 ± 0.29 mmol/L	Dietary intake (energy, protein, carbohydrate, total, fat, saturated fat, monounsaturated fat, polyunsaturated fat, alcohol, cholesterol), brain derived neurotrophic factor (BDNF), cognitive symptoms and function, cognitive speed and flexibility, cognitive flexibility
Sun (2019), China	RCT: high intensity interval training + low carbohydrate (HIIT, *n* = 18, 20.8 ± 2.7 years old), moderate intensity continuous training + low carbohydrate (MICT, *n* = 13, 21.5 ± 3.1 years old), control + low carbohydrate (LC-CON, *n* = 15, 20.9 ± 3.7 years old), vs. control (*n* = 15, 21.6 ± 3.9 years old)	4 weeks, high intensity interval training (10 × 6 s cycling sprints and 9 s recovery, total 2.5 min/session), moderate intensity continuous training (30 min cycling, 5 min for warm-up, 50% of VO_2peak_ for 10 min, 60% of VO_2peask_ for 10 min, 5 min for recovery), 5 days/week, pedometers, logbook	4 weeks, carbohydrate (10%, ~50 g/d), fat (65%), protein (~25%), urinary ketone was detected: 97.6 ± 4.5% in LC-CON, 96.2 ± 8.3% in HIIT, and 96.9 ± 6.0% in the MICT, 3-day food records	Weight, BMI, waist circumference, waist-to-hip ratio, VO_2peak_, fasting glucose, total cholesterol, HDL, LDL, triglycerides,
Perissiou (2020), United Kingdom	RCT: exercise + low carbohydrate (*n* = 33), vs. control (exercise + standard diet, *n* = 31), 35.3 ± 9 years old	8 weeks, a combination of aerobic and resistance exercise, 60%~70% heart rate (HR) peak and 85%~95% HRpeak, supervised (1st~2nd week) and unsupervised (3rd~8th week)	8 weeks, carbohydrate (>50 g/d), ß-hydroxybutyrate ketone level: >0.3 mmol/L	Cardiorespiratory fitness (VO_2peak_), Total cholesterol, HDL, LDL, triglycerides, fasting glucose, CRP, adiponectin, blood pressure, body weight, total body fat, visceral adipose tissue, lean muscle, mass, fat mass index, total bone mineral density
Freedland (2019), USA	RCT: exercise + low carbohydrate diet (*n* = 11), vs. control (*n* = 18), average 66 years old	24 weeks, ≥30 min/da, ≥5 days/week	6 months, low carbohydrate (≤20 g/day) until urinary ketone bodies detected	Carbohydrates, fat, protein, calories, weight, BMI, HOMA, glucose, insulin, HbA1c, cholesterol, LDL, HDL, non-HDL, triglycerides, prostate-specific antigen, HsCRP
LaFountain (2019), USA	Prospective intervention study: exercise + ketogenic diet (*n* = 17), 27.4 ± 6.8 years old vs. exercise + mixed diet (*n* = 17), 24.6 ± 9.0 years old	12 weeks, progressive resistance training (2 days/week, ~60 min/session, 3 sets of 12 reps at 60%-1 repetition maximum (RM) to 4 sets of 4 reps at 95%-1RM), and cardiopulmonary fitness (running, body-weight circuit training, 30 min)	Limited carbohydrate (25 g/d) and protein (90 g/d), sodium (4~5 g/d), alcohol (≥2 drinks/day), a ketone monitor: ß-hydroxybutyrate ketone level: 1.2 ± 0.3 mM	Basal metabolic rate (RMR), relative RMR, respiratory exchange ratio, % carbohydrate, % fat, anaerobic performance (1RM strength, counter movement, 10 s sprint intervals), resting metabolic rate
Jabekk (2010), Norway	RCT: exercise + ketogenic diet (*n* = 8), vs. exercise + regular diet (*n* = 8), between 20~40 years	10 weeks, resistance exercise (12 RM, supine leg press, seated leg extension, seated leg curl, seated chest press, seated rowing, seated shoulder press, seated up down, standing biceps curl)	10 weeks, restricted carbohydrates intake until urinary ketone bodies detected, urine reagent strips	Body weight, lean body mass, fasting blood lipids, glucose, dietary nutrition

## Supplementary Materials

The following are available online at https://www.mdpi.com/1660-4601/18/2/828/s1, Table S1: Assessments of the quality and risk of bias.
