# Supplementary material for: Effects of Combined Exercise and Low Carbohydrate Ketogenic Diet Interventions on Waist Circumference and Triglycerides in Overweight and Obese Individuals: A Systematic Review and Meta-Analysis
Source: Int J Environ Res Public Health. 2021 Jan 19;18(2):828. doi: 10.3390/ijerph18020828 (PMC7835865; doi:10.3390/ijerph18020828)
Supplement: Supplementary file 1 [file ijerph-18-00828-s001.pdf]

Supplementary Table S1. Assessments of the quality and risk of bias							
First Author (year)	Random sequence generation	Allocation concealment	Blinding of participants and personnel	Blinding of outcome assessment	Complete outcome data	Selective reporting	Other bias
Gregory (2017)	+	+	?	?	+	?	+
Gyorkos (2019)	+	+	?	?	+	+	+
Sun (2019)	+	+	?	?	+	-	+
Perissiou (2020)	+	?	?	?	+	-	+
Freedland (2019)	+	+	?	?	+	+	+
LaFountain (2019)	+	?	?	?	+	?	+
Jabekk (2010)	+	+	?	?	+	?	+

+ = Low risk of bias, - = High risk of bias, ? = Unclear risk of bias, ITT = Intention-to-treat
